# Advancing the future of equitable access to health care: recommendations from international health care leaders

**DOI:** 10.1093/haschl/qxae094

**Published:** 2024-08-09

**Authors:** Beth Boyer, Katie Huber, Eyal Zimlichman, Robert Saunders, Mark McClellan, Charles Kahn, Ryan Noach, Claudia Salzberg

**Affiliations:** Duke-Margolis Institute for Health Policy, Duke University, Durham, NC 27708, United States; Duke-Margolis Institute for Health Policy, Duke University, Durham, NC 27708, United States; Sheba Medical Center, Ramat Gan 52621, Israel; Duke-Margolis Institute for Health Policy, Duke University, Durham, NC 27708, United States; Duke-Margolis Institute for Health Policy, Duke University, Durham, NC 27708, United States; Federation of American Hospitals, Washington, DC 20001, United States; Discovery Health, Sandton 2146, South Africa; Future of Health, Washington, DC 20001, United States

**Keywords:** equitable access to health care, health equity, health care leaders

## Abstract

Disparities in access to health care are persistent and contribute to poor health outcomes for many populations around the world. Barriers to access are often similar across countries, despite differences in how health systems are structured. Health care leaders can work to address these barriers through bold, evidence-based actions. The Future of Health (FOH), an international community of senior health leaders, collaborated with the Duke-Margolis Institute for Health Policy to identify priority organizational and policy actions needed to improve equitable access to health care through a consensus-building exercise, a targeted literature review, and an expert discussion group. This paper describes four key action areas for health care leaders that FOH members identified as critical to enabling the future of equitable access to health care: ensuring prioritization of and accountability for equitable access to care; establishing comprehensive, organization-wide strategies to address barriers to access; clearly defining and incentivizing improvement on key measures related to reducing disparities in access; and establishing cross-sector partnerships to improve equitable access.

## Teaser

What can leaders of health organizations do to make access to health care more equitable? This paper describes four main recommendations from international health care leaders.

## Introduction

Inequitable access to health care is a persistent, growing challenge around the world, further exposed and exacerbated by the COVID-19 pandemic.^1,2^ Disparities in access to health care and health outcomes exist within all countries; however, they may vary across countries and types of health and social care systems. Even in high-income countries—of which many have adopted policies to ensure universal health coverage and address the social drivers of health—disparities remain substantial for some subpopulations.^3,4^ While specific subpopulations can vary by country, people who experience inequitable access to health care can include, but are not limited to, racial and ethnic minorities, indigenous populations, people with complex health conditions and disabilities, migrants and refugees, people with lower socioeconomic status, people who are uninsured or underinsured, rural populations, sexual and gender minority populations, justice-involved populations, and older adults. Inequitable access to *health care* is one of several contributors to inequities in *health*.^5^ People who experience inequitable access to health care often face systematic disadvantages, discrimination, and marginalization more broadly, which impacts access to economic, social, and health resources and downstream health outcomes.^6^

Despite differences in how health care systems are structured, countries share common barriers to health care access. Several frameworks conceptualize “access to care” through five domains: affordability (eg, out-of-pocket medical costs), accommodation (eg, wait times and hours for appointments), availability (eg, adequacy of provider workforce, capacity to accept new, or uninsured patients), accessibility (eg, travel times), and acceptability (eg, providers' cultural and social characteristics and beliefs).^7,8^ Broader structural and systemic barriers outside of the health care system can interact with these domains and further impact a person's ability to access health care.^6,8^ A person may experience barriers in one or multiple of these domains, and a comprehensive view of these barriers is essential to the development and implementation of strategies to achieve equitable access.

With effective leadership and innovative policies, leaders of health care delivery organizations, health insurers, and governmental health departments (“health care leaders”) and policymakers around the world can make substantial contributions to advancing equitable access to health care. This article summarizes four key action areas that international health care leaders identified as critical to advancing equitable access to care. Significant improvements could occur within the next ten years if health care leaders take bold, evidence-based actions while also ensuring ongoing accountability for improving access.

## Approach

The Future of Health (FOH), an international community of senior health leaders, collaborated with the Margolis Institute for Health Policy at Duke University to consider how health delivery organizations can advance equitable access to health care. Research activities included a review and synthesis of peer-reviewed and grey literature and qualitative analysis of two discussion groups with a total of 43 unique international health care experts (including academic researchers, health delivery organization leaders and providers, health care payers, governmental health leaders, and commercial vendors) from 12 countries in Africa, Asia, Australia, Europe, and North America to understand areas of consensus and disagreement. The first discussion group was held virtually in August 2022 with 11 participants to generate input on scoping for the topic. The second took place with 33 participants during FOH's annual summit in October 2022 in Israel. Participants completed a priority ranking exercise to identify the areas in which they as leaders could make the most impact. Participants further discussed these topics and proposed recommendations based on their experiences. For more information about meeting participants and voting process, see the Supplementary material. This paper synthesizes topics and actions FOH leaders identified as important for improving equitable access to health care, supported by findings from our review of the literature.

## Key areas for action

FOH members identified four key action areas in which health care leaders can take immediate action to improve equitable access to care in the next 10 years (Table 1).

**Table 1.** FOH member recommendations to improve equitable access to health care across four key action areas.

Action Area	Recommendations
Leadership Prioritization and Accountability	Leaders must prioritize meaningful goals related to reducing disparities and build them into their organization's mission, vision, and culture. Leaders should be accountable for driving progress on equitable access to care within the communities they serve (eg, linking measurable progress on access and outcomes to performance scorecards for board reviews; tying C-suite compensation to outcomes related to access and equity).
Designing Comprehensive Strategies to Address Disparities	Leaders should develop a comprehensive, organization-wide strategy for improving equity. The strategy should incorporate elements such as workforce trainings, increasing diversity in leadership and the workforce, patient-centered designs in services and technology, community-based care delivery models, and improvements in data quality (eg, reliable and trusted data to support measures of disparities and opportunities for improvement).
Defining and Measuring Progress on Improving Access	Leaders should undertake efforts to measure the availability and quality of health care services and track progress towards addressing barriers to access. Leaders should advocate for policy reforms to support these strategies, including the development of equity-focused payment models and national adoption of quality measures that focus on reducing disparities in access and outcomes. Leaders should implement aligned incentives to support progress and create accountability for addressing barriers in access to care.
Fostering Partnerships that Advance Equitable Access	Leaders should implement frameworks for partnerships that build on shared goals with social and community organizations. Leaders should create community-based partnerships to advance models of care in which health care and social services are extended into accessible locations in communities. Leaders should explore opportunities to establish innovative, disruptive partnerships beyond the traditional health sector.

### Leadership prioritization and accountability

FOH members believe health care leaders should make equitable access to care an explicit leadership priority for which they are held to account. Though prioritizing access is a necessary first step, it is not sufficient given that leadership and organizational priorities often change. Accordingly, leaders should be held accountable for making measurable progress towards reducing disparities in access to health care to sustain attention on long-term equitable access goals.^9^ Boards or other governing bodies should establish meaningful, measurable goals related to improving access and link progress on these goals to leaders' performance reviews and compensation to incentivize progress. For example, Jefferson Health in Philadelphia, Pennsylvania linked a quarter of the chief executive officer (CEO)'s compensation to health equity. Each year, the CEO and board set five parameters to measure progress on equity, which are directly tied to the CEO's compensation as an incentive. Elevance Health, a private health insurance provider in the US, has implemented similar steps by establishing specific performance measures for senior leadership that are linked to equity. Linking an incentive to health care access outcomes can incentivize leaders to dedicate time and attention to strategies that advance equitable access.

### Designing comprehensive strategies to address disparities

In tandem with leadership prioritization, FOH members recommend that health care leaders establish comprehensive, organization-wide strategies to address barriers to access and achieve more equitable outcomes. Leading with an intentional strategy ensures efforts are complementary, linked to a common goal, and supported by core leadership. Effective strategies should be informed by data and engagement with patients and communities. Strategies aimed at advancing equitable access to care can include workforce trainings, increasing diversity in leadership and the workforce, patient-centered designs in services and technology, community-based care delivery models, and improvements in data quality.

Examples from FOH members illustrate how such strategies can be established. For example, Henry Ford Health in Detroit, Michigan established the “On the Journey to Equity for All initiative” in 2020 to address barriers to equitable care.^10^ The plan encompasses numerous components linked to equity, including workforce trainings on implicit biases, building an inclusive and diverse workforce, expanding diversity in leadership and management roles, social advocacy, and providing platforms to learn from the community about barriers to care. Other FOH members are implementing strategies to improve access to care within targeted areas (eg, maternal health, cardiovascular disease, mental, and behavioral health), or particular interventions.^11^ Leaders should look to progress in these initial strategic areas to broaden their strategies, creating a practical path to ensure that equitable access is embedded across all areas of care delivery and that efforts are synergistic.

Throughout these efforts, health care leaders should also engage with their communities and facilitate platforms to empower and uplift voices of people who experience inequitable access to health care to advocate for policy changes that can further address systemic disparities in access to care. Leaders have the opportunity to amplify the voices and stories of people in underrepresented groups, particularly those in communities who face barriers to interfacing with health systems, which can be highly effective in influencing both organizational and policy-level changes.

### Defining, measuring, and incentivizing progress on improving access

FOH members emphasized the importance of defining clear measures of equitable access and tracking progress at both organizational and national levels. Comprehensive strategies to improve access to care require sufficient data to find and reach people who experience inequitable access to health care, as well as to track the progress and impact of efforts. At the same time, FOH members noted that standardized measures of access to care are not widely available.

Health care leaders should undertake efforts to measure the availability and quality of health care services for people who experience inequitable access to health care and track progress towards addressing barriers to access. Such efforts can help to identify barriers to care, inform strategies to address them, and track progress over time. For example, the UK's National Health Service (NHS) created a Healthcare Inequalities Improvement Dashboard to measure, monitor, and inform action on health inequities.^12^ In the US, nonprofit hospitals and local health agencies regularly complete community health needs assessments (CHNAs) and create implementation strategies to meet identified needs. Many organizations use this process to inform strategies and track progress towards improving the accessibility and quality of services. For example, Beth Israel Lahey Health in Boston, Massachusetts identified equitable access to care as a key priority in its 2022 CHNA, which informed strategies to address barriers to access and metrics to track progress, which are outlined in the system's local hospitals' implementation strategies.^13^ Policy changes to encourage CHNAs and similar efforts to measure and address disparities in access to care could expand these approaches.^14^

FOH members also recommend developing and implementing equity-focused quality measures and aligned incentives to support progress and create accountability for addressing barriers in access to care.^15^ Some countries and payers have implemented financial incentives for making measurable improvements in access to health care through pay-for-performance or more advanced value-based payment programs.^16,17^ As an example, Australia's Practice Incentives Program provides incentive payments to general practices to improve access to care, including payments for providing after-hours care, providing care in rural or remote areas, and improving care for Aboriginal and Torres Strait Islander people.^18^ Similarly, Israel introduced additional payments to incentivize health maintenance organizations to improve quality and access to primary care.^19^ Implementing measures and incentives like these can help health leaders identify disparities in access, support, and prioritize actions to address them, invest in infrastructure for ongoing assessment and quality improvement, and move towards accountability for reducing disparities.^15^

### Fostering partnerships that advance equitable access

FOH members recommend that health care delivery organizations coordinate and collaborate both within and outside of the health sector to address disparities in access to health care. Cross-sector partnerships involving diverse stakeholders can help create effective, coordinated, and sustainable solutions to drive improvements in access to care—for example, through collective impact.^20^

Health care leaders should create community-based partnerships to advance models of care in which health care and social services are extended into accessible locations in communities. For example, in the UK, integrated care systems bring together the NHS, social care providers, community- and faith-based organizations, and other partners to coordinate local services and resources to improve local health and well-being and address inequities in health access, outcomes, and experiences.^21^ The NHS also released implementation guidance for integrated care systems to engage communities in their efforts, especially people who face barriers to accessing care and people with poor experiences and outcomes.^22^ Some FOH members' organizations have also initiated community partnerships to address barriers to equitable access. For example, Jefferson Health created the Jefferson Collaborative for Health Equity, a network of partners from the health system, community- and faith-based organizations, businesses, and advocates, to address challenges related to health inequities in the Philadelphia area. The Collaborative invests in innovative solutions that make health care services more accessible while also strengthening the capacity of community organizations. Through one initiative, the Collaborative is partnering with local community health organizations to create community health care hubs, where people can access health care and social services and education.^23^

FOH members encourage identifying opportunities to establish innovative, disruptive partnerships beyond the traditional health sector, such as with technology companies, retailers, and industry to collaborate on shared goals related to equitable access to care. These stakeholders can play a significant role in shaping access to health care and social services.^24,25^ FOH members recognize that processes to form partnerships, particularly with other sectors, can be difficult to navigate. The Ottawa Hospital in Canada established an internal partnership framework guided by the Quadruple Aim to aid in developing partnerships with companies offering health-related technologies and services to improve care which may serve as an example for other groups entering partnerships. The framework allows partners to collaboratively work through their priorities, planned initiatives, roles and expectations, and expected outcomes.^26^

Moving forward, health care leaders can play an important role in driving and advocating for bold changes to create, scale, and sustain innovative partnerships that build on clear shared strategic goals. Leaders can simultaneously advocate for policy supports and incentives to facilitate effective and sustainable cross-sector collaborations.^25^ In particular, adequate funding and aligned incentives are needed to promote collaboration and sustainable activities around shared goals.

## Conclusion

Health care leaders across the FOH community firmly believe that advancing equitable access to health care is essential for redesigning the FOH. The above recommendations reflect international health care leaders' experiences and lessons and set out clear, immediate actions that other health care leaders and policymakers around the world can take to advance equitable access to health care. Improving access to health care for all paves the way for better health outcomes and a healthier population in the future.

## Supplementary Material

qxae094_Supplementary_Data
