# Supplementary material for: Advancing the future of equitable access to health care: recommendations from international health care leaders
Source: Health Aff Sch. 2024 Aug 9;2(8):qxae094. doi: 10.1093/haschl/qxae094 (PMC11332265; doi:10.1093/haschl/qxae094)
Supplement: qxae094_Supplementary_Data [file qxae094_supplementary_data.zip › Supplementary Material_HASCHOLAR-D-24-00083.docx]

**Advancing the Future of Equitable Access to Health Care: Recommendations from International Health Care Leaders**

**Supplementary Material**

**Future of Health (FOH) Members/Meeting Participants**

**Supplementary Table A1.** Participants in August 2022 Scoping Meeting by Country and Organization

| **Country** | **Organization** | **Name** |
| --- | --- | --- |
| **Canada** | The Ottawa Hospital | Alan Forster |
| **South Africa** | Discovery Health | Roseanne Harris |
| **Switzerland** | University of Zurich | Nikola Biller-Andorno |
| **United Kingdom** | National Health Service | Lucy Dadge |
| **United States** | University of Chicago | Marshall Chin |
|  | Commonwealth Fund | Munira Gunja |
|  | Centers for Medicare & Medicaid Services | Dora Hughes |
|  | Community Care Cooperative | Philly Lapiste |
|  | Community Care Cooperative | Joe Mando |
|  | Henry Ford Health | Alex Plum |
|  | University of California San Francisco | Mark Smith |

*Note: Affiliations at time of meeting*

**Supplementary Table A2.** Participants in October 2022 Voting Meeting by Country and Organization

| **Country** | **Organization** | **Name** |
| --- | --- | --- |
| **Australia** | Sir Peter MacCallum Cancer Centre | Shelley Dolan |
| **Bahrain** | American Mission Hospital | George Cheriyan |
| **Canada** | Centre Hospitalier de l’Université de Montréal | Fabrice Brunet |
|  | The Ottawa Hospital | Alan Forster |
|  | Integrated Health and Social Services University Network for West-Central Montreal | Lawrence Rosenberg |
| **Denmark** | Danish Regions | Erik Jylling |
| **France** | North-University of Paris Hospital Group | Vincent-Nicolas Delpech |
| **Germany** | Charité – Universitätsmedizin Berlin | Martin Kreis |
|  | Charité – Universitätsmedizin Berlin | Heyo Kroemer |
| **Israel** | Sheba Medical Center; Israel Foundation for Crohn’s Disease and Ulcerative Colitis | Adam Elgressy |
|  | Sheba Medical Center | Yitshak Kreiss |
|  | Israeli Ministry of Health | Osnat Luxenburg |
|  | Sheba Medical Center | Eyal Zimlichman |
| **South Africa** | Discovery Health | Ryan Noach |
| **Sweden** | Karolinska University Hospital | Bjorn Zoega |
| **Switzerland** | University Hospital Zurich | Christoph A. Meier |
| **United Kingdom** | Retired health leader | Bob Bell |
|  | Microsoft | Umang Patel |
|  | Great Ormond Street Hospital | Andrew Taylor |
| **United States** | Brigham & Women’s Hospital | David Bates |
|  | Cincinnati Children’s Hospital | Steve Davis |
|  | Northwestern Medicine | Daniel Derman |
|  | Henry Ford Health System | Scott Dulchavsky |
|  | Brigham and Women’s Hospital | Sunil Eappen |
|  | Stanford Medical School | Peter Fitzgerald |
|  | Federation of American Hospitals | Chip Kahn |
|  | CommonSpirit Health | Gary Kaplan |
|  | General Catalyst | Stephen Klasko |
|  | Advisory Board | Eric Larsen |
|  | Advisory Board | Vidal Seegobin |
|  | BD | Joseph M. Smith |
|  | Beth Israel Lahey Health | Kevin Tabb |
|  | General Catalyst | Daryl Tol |

*Note: Affiliations at time of meeting*

**Ranked Choice Voting Process**

In the October meeting, participants were asked to consider action areas that should be prioritized to improve equitable access to care. They were presented with six options derived from the previous expert meeting and a review of the literature. These action areas included:

1. Establishing Equitable Access to Care as a Leadership Priority
2. Improving the Availability and Quality of Health Data for Medically Underserved Populations
3. Building Workforce Capacity to Facilitate Broader Availability and Cultural Appropriateness of Care
4. Building Cross-Sectoral Partnerships to Expand Access to Health and Social Care
5. Designing Accessible Community-Based Models of Care
6. Providing Incentives for Health Care Transformation to Advance Equitable Access

Participants selected action areas 1, 4, and 6 as their top priorities. The participants were then asked to vote on sub-priorities within each of these action areas. Each round of voting was supplemented with discussion with the members to support their choice or defend an option that did not receive majority vote. The key areas for action identified in our paper arose from a combination of these voting exercises and the discussion that followed.
